# *STAT5b* in LGL leukemia – a novel therapeutic target?

**DOI:** 10.18632/oncotarget.1035

**Published:** 2013-05-16

**Authors:** Hanna L. M. Rajala, Satu Mustjoki

**Affiliations:** Hematology Research Unit Helsinki, Department of Medicine, University of Helsinki and Helsinki University Central Hospital, Helsinki, Finland; Hematology Research Unit Helsinki, Department of Medicine, University of Helsinki and Helsinki University Central Hospital, Helsinki, Finland

The activation of signal transducer and activator of transcription (STAT) family genes, especially *STAT5* and *STAT3*, has been established in multiple solid tumors and hematological malignancies. Recently we discovered somatic activating *STAT3* mutations in 40% of T-cell large granular lymphocytic (LGL) leukemia and 30% of NK-cell LGL leukemia patients (Koskela, Eldfors et al. NEJM, 2012, 17;366(20):1905-13 and Jerez et al. Blood, 2012, 120(15):3048-57). Interestingly, our current findings indicate that also activating *STAT5b* mutations can be found in LGL leukemia patients (Rajala et al. Blood Apr 17, 2013).

LGL leukemia is incurable disease, characterized by chronic expansion of cytotoxic T or NK cells, and it often manifests with other hematological and autoimmune disorders such as rheumatoid arthritis. Autoimmune mediated cytopenias such as neutropenia are common and can be life-threatening due to increased infection-susceptibility. Leukemic T-LGLs have a phenotype of terminally differentiated effector-memory cells and, accordingly, the role of viral antigens or autoantigens as potential drivers of the clonal expansion has been speculated. However, in leukemic LGL cells the normal activation-induced cell death (AICD) is impaired, and several cell-survival signaling pathways such as JAK-STAT, MAPK/ERK/Ras, PI3K-AKT, and NfκB are activated. Common immunosuppressive agents such as prednisone and methotrexate are current first-line treatments, and no targeted therapies exist.

By means of whole exome sequencing we identified a *STAT5b* mutation Y665F in two *STAT3* mutation-negative LGL leukemia patients, and in the following screening of a large patient cohort (n>200) another *STAT5b* mutation N642H in two additional patients. Both these mutations are located in the src-like homologue 2 (SH2) domain of STAT5b. No mutations were found in *STAT5a*, which shares over 90% of similarity with *STAT5b* in cDNA level. SH2 domain plays a crucial role in STAT5b activation and facilitates the dimerization of phosphorylated STAT5b monomers and their subsequent translocation to the nucleus where they activate the transcription of downstream target genes by binding to consensus DNA motifs. STAT5 is important in normal lymphocyte development, and in a mice model *STAT5a/b* double-deficiencyleads to perinatal death and severely impaired development of lymphoid tissue. In humans, homozygous germline *STAT5b* mutations are linked with disturbed T and NK cell homeostasis in addition to postnatal growth failure due to growth hormone-insensitivity.

**Figure 1:**
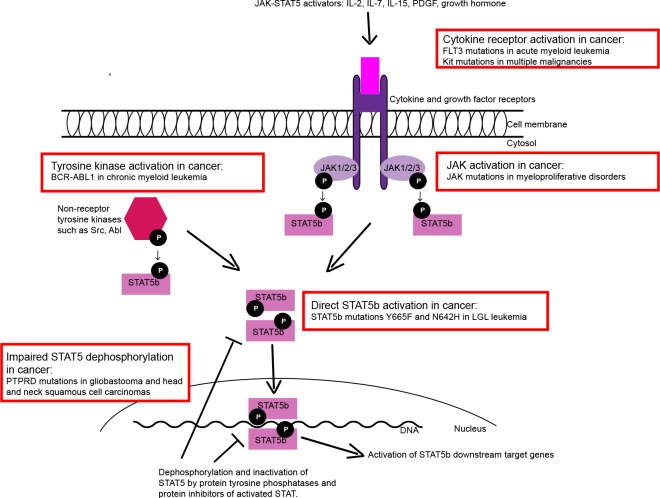
Different mechanisms of STAT5 activation in cancer STAT5 is activated and phosphorylated by different cytokine receptors (through JAK) and non-receptor tyrosine kinases. Phosphorylated monomers form dimers due to reciprocal SH2-pY interaction, translocate to the nucleus and bind to target DNA. The STAT5 activation is abrogated by tyrosine phosphatases and protein inhibitors. Different mechanisms of STAT5 activation in cancer are shown as examples (red boxes).

STAT5 is constitutively activated in many human malignancies such as in breast and prostate cancer, glioblastoma, acute myeloid leukemia (AML), myeloproliferative disorders and chronic myeloid leukemia (CML). In these conditions STAT5 activation is hypothesized to be a secondary event caused either by overly active upstream pathway or defective dephosphorylation and inactivation of STAT5 (Figure 1). For example in CML, the STAT5 activation is induced by oncogenic BCR-ABL1 fusion protein, and the silencing of STAT5 in BCR-ABL1 expressing cells triggers apoptosis.

Our results in LGL leukemia patients demonstrate a novel mechanism of STAT5 activation in cancer: somatic point mutations (Figure 1). Based on the three-dimensional model of STAT5, we speculate that the mutations located in the SH2 domain stabilize the dimer structure, thus inclining the balance of STAT5 activation-deactivation toward the constitutively active dimer form. In concordance with this theory, our results showed that the *STAT5b* mutations Y665F and N642H increased the phosphorylation and transcriptional activity of STAT5. Furthermore, in an earlier *in vitro* study in which the mutation STAT5b N642H was discovered by random mutagenesis, it caused cytokine-independent growth of mouse Ba-F3 cells and hyperphosphorylation of STAT5 after IL-3 stimulation. Similarly in LGL leukemia, we discovered increased phosphorylation of STAT5b, but it was only observed in the patients with the STAT5b mutations. This differs from STAT3 phosphorylation, which is universally seen in leukemic LGLs. However, there are intriguing connections between STAT3 and STAT5 downstream pathways. Recent publication presenting ChIP-seq data from T-cells showed that 90% of STAT5 binding sites are co-occupied with STAT3. This is in accordance with our results demonstrating that LGL leukemia patients with *STAT3* and *STAT5b* mutations share similar gene expression patterns.

Although LGL leukemia is not always life-threatening, a majority of patients need treatment due to cytopenias or autoimmune manifestations. Importantly, the two patients who had somatic STAT5b N642H mutations suffered from a rare, rapidly-progressing chemorefractory form of LGL leukemia. They were the only ones with the aggressive type of the disease in the patient cohort screened (n>200). Thus, it is likely that this mutation is a driver mutation in this type of aggressive LGL leukemia and it is tempting to speculate that targeted STAT5 inhibition may have clear therapeutic potential. Currently, specific STAT5 inhibitors are not in clinical use, but they are under development. One non-specific STAT5b inhibitor, pimozide, has been shown to induce apoptosis through BCR-ABL1-independent STAT5 inhibition in CML cells and also in FLT3-mutated AML model. Other promising therapeutic agents include small-molecule inhibitors, which bind directly to the STAT5 SH2 domain and prevent phosphorylation and dimerization required for STAT5 activation. In a previous study, which tested a series of chromone-derived acylhydrazone compounds, STAT5 inhibition was demonstrated in Daudi-cells, and in another study similar effects were seen with SH2 domain-binding salicylic acid-containing inhibitors.

Our discovery of *STAT5b* and *STAT3* mutations suggests novel therapeutic targets for LGL leukemia. Recently similar *STAT3* mutations have been discovered in a proportion of CD30+ T-cell lymphoma patients and in inflammatory hepatocellular adenomas, but so far *STAT5b* mutations have only been found in LGL leukemia patients. However, as STAT5 activation is an important secondary event in many cancers, the targeted STAT5 inhibition may have a clear therapeutic potential in the future.

